# Assessment policy of post-traumatic stress disorder in aviation and its practical application using turbulence-triggered trauma as an example

**DOI:** 10.3389/fpubh.2025.1505004

**Published:** 2025-03-18

**Authors:** Alpo Vuorio, Robert Bor, Bruce Budowle, Alastair Gray, Anna-Stina Suhonen-Malm

**Affiliations:** ^1^Department of Forensic Medicine, University of Helsinki, Helsinki, Finland; ^2^Mehiläinen Airport Health Centre, Vantaa, Finland; ^3^Centre for Aviation Psychology, London, United Kingdom

**Keywords:** aviation, cabin crew, pilot, mental health, post-traumatic stress disorder, turbulence, International Classification of Diseases, Federal Aviation Administrator

## Abstract

The U.S. Federal Aviation Administration (FAA) recently provided detailed instructions on how Aviation Medical Examiners (AMEs) should assess and evaluate pilots for post-traumatic stress disorder (PTSD). The European, Australian and International Civil Aviation Organization guidelines for the assessment of PTSD in aviation are general guidelines and do not address the unique and specific circumstances of a flight crew *per se*. The starting point of the U.S. FAA’s guidance is an already-established clinical PTSD diagnosis since it is known that PTSD compromises aviation safety and has been related to fatal aviation accidents. According to the FAA’s guidance, a PTSD assessment is undertaken based on whether the condition is symptomatic and medicated, or whether more than 2 years have elapsed since showing symptoms and receiving medication. The International Classification of Diseases (ICD) criteria for stress disorders have changed between versions ICD-10 and the soon-to-be-released ICD-11. The new ICD-11 criteria are discussed in this article in the context of aviation health. Additionally, PTSD, potentially caused by an incident of turbulence, is discussed in the context of aviation mental health. There are currently no published studies on turbulence-caused mental trauma. We have identified in this article potential factors which are related to pilots’ and cabin crew’s stressors in incidents of severe and extreme turbulence. Three recommendations are provided: (1) harmonize assessment practices of PTSD internationally; (2) healthcare professionals taking care of traumatized flight crew should have a follow-up guide that takes specific and local conditions into account, and ensures the identification of patients who require follow-up treatment as early as possible; and (3) aviation health care professionals should consider ICD-11 diagnostic criteria as the information may be more useful in the assessment and diagnosis of aviation-related trauma.

## Introduction

There is a well-recognized risk among aviation personnel, particularly pilots, cabin crew and air traffic controllers, of being exposed to traumatizing incidents related to flying. Practical and emotional support following traumatic events have been implemented at a practical level although debriefings are rare as this may impede speedy psychological recovery (1). According to the UK Civil Aviation Authority, individuals exposed to critical incidents should be immediately withdrawn from duty (2). Those individuals need time to rest, be available for medical examination if needed, have time to complete the necessary reports and be available to assist the investigating team. What constitutes a “critical incident” and determining its impact and severity on an individual will need to be clinically determined in each case.

Longer-term mental health effects caused by trauma are well known. However, there is a lack of longer-term follow-up studies showing the risk of developing post-traumatic stress disorder (PTSD) and related mental health conditions and symptoms following the specific context of aviation incidents and accidents, which by definition may impact a much wider number of people. A 1995 study of 6 cabin crew members in a turbulence-related aircraft accident in which 47 passengers were killed (3) reviewed cabin crew mental health interviews carried out between 8- and 10-months post incident. The study reported that all cabin crew members met the Diagnostic and Statistical Manual of Mental Disorders Third Edition Revised (DSM-III-R) diagnostic criteria for PTSD (the prevailing mental health classification system at the time) (4). All crew members subsequently developed a fear of flying, and they all also reported a fear of turbulence. The most intense and severe symptoms were reported by the most senior members of the cabin crew who had also suffered physical injury.

In 2009, an aircraft crashed near Amsterdam (5). Most adult survivors of the accident (passengers and crew) were followed up using the Trauma Screening Questionnaire at 2 and 9 months after the accident (6). A questionnaire was administered to 121 survivors including crew members and passengers. This study found at 2 months 46% and at 9 months 47% of participants were at risk of PTSD. At 2 months post-accident, there were 39 non-responders and at 9 months 45 non-responders in this follow-up study representing a significant rate of subject withdrawal from the study.

PTSD has been shown to be a risk factor for aviation safety (7). In this article, we discuss the assessment policy after exposure to trauma in aviation. Of particular interest is the U.S. Federal Aviation Administration’s (FAA’s) protocol that was introduced in June 2024 for assessing PTSD in pilots (8). In the article herein, we consider the applicability of this new FAA protocol in assessing the fitness to work/operate aircraft by aircrew experiencing stress-related symptoms after significant turbulence.

The purpose of this Policy and Practice Review is to raise awareness of the updated diagnostic criteria for PTSD that differs between the ICD-10 (International Classification of Diseases diagnostic system) and the new ICD-11 system. Additionally, PTSD aviation medicine assessment protocols provided by different aviation authorities are compared. Furthermore, the potential role of turbulence as a trigger for PTSD is discussed. We also consider why there is a need to follow-up with flight crew after such incidents. To the best of the authors knowledge there are no published studies on this topic in aviation medicine.

## Methods

Literature searches were carried out in August 2024 via the Medline and Cochrane databases to identify relevant research articles, reviews, or systematic reviews focusing on PTSD, ICD-10, ICD-11, and fatal Aviation Accidents. The medical requirements of different aviation authorities from the U.S. FAA, Australia (Civil Aviation Safety Authority), Europe (European Aviation Safety Agency), and the International Civil Aviation Organization (ICAO) Manual of Civil Aviation Medicine were examined and compared using information from the websites of these organizations. The data relating to recent turbulence accidents were based on accident reports and relevant newspaper articles.

## Trauma-related stress disorder in the International Classification of Diseases diagnostic system

The Diagnostic and Statistical Manual of Mental Disorders (DSM), published by the American Psychiatric Association (APA), is used in the U.S. and in other jurisdictions to diagnose and classify psychiatric symptoms and conditions (9). The International Classification of Diseases (ICD), produced by the World Health Organization (WHO), is used in Europe (10). The current versions in use are DSM-5 and ICD-10, respectively, which are revised and updated periodically. DSM and ICD systems diagnostic criteria differ in some aspects (Table 1). For example, in the forthcoming update from ICD-10 to ICD-11, there is a narrower concept for PTSD, and both acute stress reaction and acute stress disorder will no longer be diagnostic entities while they are still present in the older ICD-10 and DSM-5 (10, 11). The appropriateness of the ICD-11 diagnostic criteria for stress related disorders in clinical practice has been studied to some extent by Ederle & Maercker (12). This study showed that stress-associated disorders, as defined by ICD-11, have discrete features typical for each diagnostic group. Furthermore, it was shown that in each diagnostic group, cognitive thinking styles, such as preoccupation, were associated with flashbacks and rumination. Additionally, Møller et al. (13) studied how well ICD-11 diagnoses of PTSD overlap with ICD-10 diagnoses. In their study of 165 Danish psychiatric outpatients they found that 23% with ICD-10 PTSD did not meet the ICD-criteria for a PTSD diagnosis. This finding is supported by other studies (14, 15). The result is somewhat expected because ICD-11 diagnostic criteria focus on core post-traumatic responses and should potentially reduce overlap with depression and anxiety (13). The results are mainly based on psychiatric data obtained from psychiatric patients. It remains to be seen if this change in diagnostic criteria affects PTSD diagnosis in aviation medicine.

**Table 1:** Comparison of International Classification of Diseases (ICD) systems (10, 47).

Disorder	ICD-10	ICD-11
Acute stress reaction	Occurs within minutes of the triggering event and resolves rapidly if the stressor is removed, and within 1–3 days even if it is not.	No longer a diagnostic entity.
Acute stress disorder*	Symptoms begin within 4 weeks of the trauma and last a minimum of 3 days, but no more than 1 month.	No longer a diagnostic entity.
Posttraumatic stress disorder*	Experiencing threatening or catastrophic event Intrusive flashbacks Avoidance Changes in cognition and mood Either A or B: A. Inability to recall, either partially or completely, some important aspects of the period of exposure to the stressor. B. Persistent symptoms of increased psychological sensitivity and arousal All criteria must last several weeks and met within 6 months of the stressful event.	Experiencing an extreme threatening event Re-experiencing Avoidance Persistent perceptions of heightened current threat The symptoms must last for at least several weeks. Significant functional impairment.
Complex posttraumatic stress disorder	Not covered	Experiencing an extreme threatening event. Characterized by the core symptoms of PTSD as well as persistent impairments in affective, self- and relational functioning, difficulties in emotion regulation, beliefs of being worthless, difficulties in relationship.

*Are diagnostic terms in Diagnostic and Statistical Manual of Mental Disorders, Fifth Edition (DSM-5) (48).

ICD-10 is used as the diagnostic basis for ASR (acute stress reaction), ASD (acute stress disorder) and PTSD. Additionally, potential new challenges are discussed relating to new diagnostic criteria of stress disorders prescribed by the new ICD-11 in the context of flight personnel aeromedical assessment. Stress diagnoses defined in ICD-11 associated with prolonged stress such as prolonged grief disorder are also covered herein.

### PTSD in the diagnostic and statistical manual of mental disorder diagnostic classification

It is noteworthy that DSM-5 has taken a broader approach to PTSD compared to that of ICD-11. In addition to core symptoms of PTSD intrusions, avoidance and arousal activity, DSM-5 includes symptoms related to mood changes such as negative beliefs, having distorted thoughts, negative emotions, reduced interest in significant events and feeling detached. Supplementary Appendix 1 lists PTSD diagnostic criteria based on DSM-5.

### Prolonged grief disorder

A new diagnostic disorder in ICD-11 associated with stress is prolonged grief disorder (10, 11). The diagnosis describes abnormally persistent grief. It is defined as a persistent and pervasive yearning for the deceased following the death of a person close to the bereaved, or a persistent preoccupation with the deceased. Symptoms extend at least 6 months and cause significant impairment in the person’s functioning. With this disorder symptoms of grief go far beyond a person’s cultural context. The evidence for this new diagnosis comes from a variety cultures (16). In addition to grief, these patients have mental health problems associated with suicidality, substance abuse and harmful health behaviors and cognitive dysfunctions (16, 17). Prolonged grief disorder also has been introduced in DSM-5-TR, and there are some differences compared to ICD-11 diagnostic criteria (18). This disorder is discussed briefly herein because it is new and potentially related to flight crew health assessment.

## Assessment protocol for PTSD by different aviation authorities

### Assessment protocol for PTSD in Aviation by International Civil Aviation Organizations, Civil Aviation Safety Authority Australia and European Union Aviation Safety Agency

The European, Australian and International Civil Aviation Organization guidelines for the assessment of stress reactions in aviation are general guidelines and do not address the unique circumstances of flight crew *per se* (Table 2) (19–21). The assessment of PTSD has not been elevated to a special status in these guidelines, and the scope of the assessment remains largely discretionary.

**Table 2:** Guidance Provided by ICAO, CASA Australia and EASA of the Assessment Stress Related Disorders (19–21).

Authority/Organization	Guidance
ICAO	Psychiatric symptoms due to external stressors leads to being temporarily unfit. Reassessment after a period of stability without use of psychotropic medication. Note alcohol and/or other psychoactive substances use.
CASA Australia	General assessment for all psychiatric disorders: Symptoms, medications, recurrence, risk of harm to self and others and comorbidities and organic causes.
EASA	Signs or evidence that applicant has stress-related disorder, then needed psychologic or psychiatric opinion and advice

### Assessment protocol for PTSD in aviation by the U.S. FAA

The FAA recently provided detailed instructions on how Aviation Medical Examiners (AME) should assess and evaluate pilots for PTSD disorder. These very detailed instructions are unique among air medicine guidance. As mentioned above, it is typical for authorities to instruct an evaluation on a general level, whereas the starting point of the FAA’s guidelines is an already-established PTSD diagnosis (8). In such cases, the evaluation is performed differently based on whether PTSD is symptomatic and medicated, or whether more than 2 years have elapsed since symptoms were identified and medication started. In the latter case, if there have been no symptoms or medication for 2 years, the AME can make an assessment independently (Table 3).

**Table 3:** FAA U.S. assessment protocol for PTSD (8).

	AME	FAA
Condition	No PTSD symptoms and no SSRI* or other psychiatric medications in the past 2 years	PTSD symptoms and/or SSRI or other psychiatric medications
Evaluation	Information regarding diagnosis severity treatment symptoms fill in *Anxiety, Depression, and Related Condition Tool for the AME*	*Airman statement covering* incident(s) triggers and symptoms impact for life modifications to work medications any form of counseling *Current evaluation psychiatrists/psychology* *Medication list* *Copies of PTSD screening tools*
Assessment	AME may assess	FAA will assess

*SSRI, selective serotonin reuptake inhibitors.

The FAA’s guidelines place more weight on previous mental health symptoms. To evaluate these conditions, a pilot-completed questionnaire has been devised (Table 4) (22). If a positive answer is provided to any items on the questionnaire, decisions regarding fitness to fly are immediately transferred to the crew licensing authority for further evaluation.

**Table 4:** Anxiety, depression, and related conditions decision tool for the AME (22).

Questions to be considered
Any other mental disorder diagnosis or symptoms?
History of suicide or self-harm?
History of involuntary mental health condition or substance use?
History of electroconvulsive, transcranial magnetic, ketamine, or psychedelic therapy?
History of psychiatric hospitalization?
Individual experienced more than one episode?
Continued symptoms severe enough to interfere with safety?
Mental health medications?
Is AME having any concerns?

## Practical examples of aircraft accidents and stress/PTSD

Trauma follows from an event that poses a significant challenge to the emotional and/or physical safety of person involved in the situation, whether directly or indirectly exposed to the triggering situation or event (23). Regarding aviation, a traumatic experience comprises in-flight events and as well as events related to the rescue operation. It has been shown in studies in which victims of aviation-triggered PTSD were followed up, the risk factors related to this disorder were loss of relative in the aircraft accident, the severity of physical symptoms and the severity of the event itself (24).

In an accident involving SK751 flight on 27 December 1991 from Stockholm to Warsaw 129 persons onboard who all survived, the aircraft MacDonnell Douglas MD-80 rapidly descended due to a loss of engine power and some minutes later crashed onto a field. An opportunistic, follow-up study of PTSD among those people on board was carried out (25). There were initially high levels of PTSD. Most changes in the presence of PTSD symptoms occurred between 1 and 4 months and after that there was only a small decrease occurred from 4 to 25 months.

### Turbulence related incident and stress

In-flight turbulence may be a specific trigger trauma and PTSD. In the recent analysis medical data, which compared turbulence events between 2019 and 2023, it was shown that there was an increase in turbulence-related injuries over this period and that about 10% of these incidents caused acute stress reactions (26). The analysis involved specifically business aviation and found that that in 2019, 123 cases of injury and in 2023, 187 cases of injury attributed to turbulences. Among these cases, there were 47 and 53 crew members in 2019 and 2023, respectively, injured due to turbulence. These incidents can trigger PTSD in those injured in such circumstances.

The recent incident from commercial aviation will be introduced. On the 21st of May 2024 Singapore Airlines flight SQ321, carrying 211 passengers and 18 crew members, was involved in a serious turbulence incident that resulted in 70 injuries and one death (27). The initial report by the National Transportation Safety Board of Singapore mentioned that the first vertical acceleration decreased from +1.35 G to −1.5G resulting in those cabin crew and passengers who were not wearing seat belts becoming airborne. Vertical acceleration then changed from −1.5 G to +1.5 G resulting in those same passengers and crew falling back down, resulting in severe injuries (28).

The paucity of studies is noted about the mental symptoms of pilots and cabin crew after highly stressful events caused by turbulence that negatively affect both their physical and psychological health. In addition to symptoms related to short term acute stress reactions, mental and physical traumas related to serious incidents like turbulence, may potentially be associated with an increased risk of developing PTSD (29, 30). Our current understanding of the PTSD risk in flight crew is based on limited studies of aircraft crash accidents (3, 5).

A National Transportation Safety Board (NTSB) analysis of commercial aircraft operations from 2009 through 2018 found that turbulence-related accidents were among the most common types of accidents accounting for one-third of incidents and accidents recorded and their number is increasing (31–34). According to ICAO’s 2024 Annual Safety Report, turbulence-related accidents were around 40% of all accidents involving large aircraft (34). In addition, most turbulence-caused accidents resulted in one or more serious injuries (33). The NTSB report offers flight-safety-related technical operational recommendations as well as new recommendations for cabin crew pertaining to the phases of flight and altitude at which cabin crew should be secured in their seats. Additionally, the report recommends improving in-flight communication between pilots and cabin crew.

Most of the turbulence-related incidents reported to the NTSB were moderate or severe. During moderate turbulence, passengers may feel strain against seat belts while during severe turbulence passengers are violently forced against their seat belts (assuming that they are “strapped in”). During extreme turbulence, the aircraft may be impossible to control, passengers may suffer severe injuries, and the aircraft may undergo structural damage. In the above-mentioned NTSB report, there were 97 seriously injured cabin crew members, and over 80% of them were not using seat belts when turbulence occurred (33). Most serious injuries were related to the lower and upper extremities and spine.

While there are no studies currently available on turbulence-caused mental trauma, there are potential stressors that could be key predictors in identifying air crew mental health issues due to being exposed to severe turbulences (Table 5). The type and strength of stressors are different among cabin crew and pilots and depends on the incident type. Furthermore, immediate post-incident stress could be exacerbated by reports and incident investigation, which in turn may impact upon pilots and cabin crew differently.

**Table 5:** Potential turbulence incident related acute stressors among pilots and cabin crew.

Turbulence type	Pilots’ stressors	Cabin crew’s stressors
Severe	Aircraft control Application of protocols and sufficiency of crew-resource Acute situation in cabin	Own injuries Behavior of colleagues and their injuries Passengers’ behavior and their injuries
Extreme	Own injuries Aircraft control due to damage and sufficiency of crew-resource management Flight protocols not applicable Acute emergency in cabin	Own injuries Behavior of colleagues/pilots and their severe injuries and possible deaths Passengers’ behavior and their severe injuries and deaths

From the aviation medicine assessment perspective, there is the need for acute support, followed by assessments relating to when pilots and cabin crew are fit to return to duty. This should be followed by timely follow-up with longer-term assessments completed due to the risk of developing PTSD. The U.S. FAA recommends that PTSD assessment should be performed earlier when dealing with significant mental health history as it can be an important risk factor for PTSD (8). A recent systematic review of road accidents found that female sex, sociodemographic markers, pre-trauma, peri-trauma, and post-trauma factors were predictors of PTSD (35). PTSD was assessed between 2 weeks to 3 years post-incident. The significant predictors of PTSD were being female, pre-existing depression, previous road traffic accidents, peritraumatic dissociative experiences, acute stress disorder diagnosis, rumination, injury severity, and involvement in litigation after trauma. While these results are derived from studies of road accidents, they could be applicable to risk-profile assessments of pilots and cabin crew following turbulence-related incidents. In this situation, it is not enough for aviation occupational health care specialists to identify the immediate factors related to the trauma itself (although they also need to be carefully identified); it is important to understand the diverse risk factors underlying the risk of PTSD.

### Traumatic incident in the aviation occupational healthcare

When using the ICD-10 diagnostic criteria, PTSD diagnosis is usually performed after approximately 6 months but can be performed as early as 1 month. A common challenge, based on clinical experience, is that it is not easy to keep the crew under close supervision after an incident. To be able to assess the occurrence and development of possible symptoms experienced by the crew, it would be helpful if a structured interview related to the symptoms of trauma was conducted for every member of the crew exposed to a significant stressor. These structured interview templates would be available to suit different diagnostic systems (36–38). There is no uniform practice regarding the timing of assessments and structured interviews after aviation accidents. However, it is important especially regarding the flight crew that they cover the early stages, and that follow-up includes a patient appointment before returning to duty. Based on the results of the first assessment and the structured interview it is important to schedule the follow-up assessments including the interviews. The second assessment and interview are recommended to be scheduled within 3 months because trauma symptoms during recovery from PTSD following acute trauma exposure according to systematic review occur mainly during the first 3 months after trauma (39). The follow-up needs to be more in depth and longer lasting for crew members with an increased risk of PTSD as well as with those that are symptomatic. Table 6 presents the practical functions of aviation occupational health care in the treatment of aviation mental trauma. Especially continuous education is utmost important since the EASA MESAFE Report has shown that some AMEs may have insufficient experience regarding psychological and psychiatric assessments (30). This survey revealed that almost half of the respondents do not have consistent and effective criteria to decide whether to refer a pilot to a mental health specialist.

**Table 6:** Practical functions of aviation occupational health care in the treatment of aviation mental trauma.

Function	Practical action
The role of airline providing acute psychological support	Advance preparation and agreement on consultation readiness with clinical/aviation psychologists and psychiatrists
Long-term support	Systematic monitoring of trauma symptoms through structured interviews, especially during the first 4 months after accident
Continuous education	Identification and treatment of trauma symptoms associated with aviation accidents using appropriate consultations
Accidents/incidents	Because accidents/incidents may happen anywhere in the world, the airline must create an international cooperation plan.
Periodical medical examinations*	Ask whether the person being examined has been exposed to accidents/incidents

*Periodical medical examinations are carried out for airline pilots at least once a year but for cabin crew, varying depending on the country. In Europe examination is carried out for cabin crew at least every 5 years but f. ex. in the U.S. only when starting work.

## Discussion

There is limited evidence that PTSD presents itself in some fatal aviation accidents (3, 7). We found in the NTSB accident database between 2000 and 2015 eight pilots with a diagnosis of PTSD, two of whom had aviation-related PTSD. Also, the lack of follow-up studies after traumatizing incidents was noted. Additionally, these studies showed that pilots did not inform their AME or employer of their pre-existing mental symptoms. Currently, there is a lack of research on PTSD as a risk factor for aviation accidents (40). There is a culture within aviation that not to report mental health related symptoms to AME because of fear losing career and medical certification. There is also existing a distrust in the aviation authority medical assessment and much of this distrust is due to lack of information about the aviation authority processes (41).

Aviation health regulators have until recently dealt sparingly with pilot PTSD assessment, and the new U.S. FAA protocol is welcomed. Considering the concern highlighted herein, there is an urgent need to address PTSD further and on an international basis. Whether there is a need for detailed PTSD-specific assessment protocols or more formal adoption of more frequent cycles of general mental assessment (to include mental health risk analysis) with PTSD needs to be discussed. There is a great need to increase awareness of PTSD as a potential disorder affecting flight safety. With the frequency of serious turbulence incidents predicted to rise, in line with climate change, it is reasonable to conclude that the rates of mental trauma experienced by air crew because of these changes in climatic conditions are going to increase (42). The evaluation of cabin crew has not been required until now, and the protocols for doing so are not clearly articulated. The current situation requires more detailed instructions for the cabin crew. PTSD diagnostic criteria are changing, and the ICD-11 system will be introduced in the near term. Aviation occupational health units need to take account of this change in their training. It is also worth considering that as part of ICD-11 there are new diagnoses such as prolonged grief disorder and complex PTSD.

There is a growing literature using new methodologies to study PTSD. For example, there are studies using functional MRI and neural network modeling to research subtypes of PTSD (43, 44). It is possible that these new results will offer improvement in diagnostics as well as new and specific options for the treatment of PTSD. Better diagnostics (40) and new therapeutic methods (45) should allow even more air personnel to return to work after severe traumatic events. In neural contributors to trauma resilience, neuroimaging studies have shown individual differences in neural function that appear protective against the psychopathology following a major life stressor (46).

As already mentioned, there is a specific lack of studies about the mental symptoms of pilots and cabin crew after highly stressful events caused by turbulence, or indeed related traumatic ground- or in-flight untoward and potentially traumatic events. In this review, we have identified potential factors that are related to pilots’ and cabin crew’s stressors in incidents of severe and extreme turbulence and potentially are triggers of PTSD. Medical and psychological follow-ups of aircrew several months after severe turbulence are necessary to identify those pilots and cabin crew members who manifest PTSD. PTSD is harmful to an individual, but it also may compromise flight safety. Returning to flight duty after PTSD needs careful assessment. The assessment protocol provided by the U.S. FAA is a welcomed addition, but it is recent, and there is still little experience in its practical application. Even though data are scant, it is well-accepted that trauma can trigger PTSD. The intention of this article is to raise awareness to motivate aircraft pilots and crews, investigators, healthcare practitioners, and researchers to consider turbulence as another potential trigger in their assessments, gather more data and continue to develop policies and protocols to improve aviation safety.

## Findings

1. Aviation personnel are at increased risk of being exposed to traumatizing incidents associated with ground operations and actual flight.
2. Aviation personnel risk developing PTSD which compromises personal well-being and potentially flight safety. It also may lead to attrition in aircrew.
3. The diagnostic criteria for PTSD are currently changing because of modifications from ICD-10 to ICD-11.
4. Different aviation authorities have differing medical assessment protocols for pilots diagnosed with PTSD.
5. Turbulence-related incidents appear to be increasing in frequency.
6. Severe turbulence may be a trigger for PTSD although there is a lack of research studies to support this position.
7. There is a need to follow-up aviation personnel after severe turbulence and to create a protocol for this purpose.

## Recommendations

1.  The U.S. FAA has recently prepared in detail an assessment protocol for PTSD.

  Harmonize international assessment practices of PTSD in aviation medicine so that aviation authorities throughout the world can prepare assessment protocols for PTSD that are based on upon consistent criteria. A similar type of protocol can best ensure that the assessment and monitoring of flight crew performance are carried out routinely, effectively and with adequate comparison. This protocol should also include a recommendation on international cooperation in acute situations.

2  Follow-up guidance should be issued for healthcare professionals who take care of traumatized flight crew that also takes local conditions into account, which ensures the identification of patients who require follow-up treatment as early as possible.
3  Healthcare providers should move from ICD-10 diagnostic criteria to those in the ICD-11 diagnostic criteria. There are differences between the criteria in each document regarding acute and long-term diagnostic criteria. Aviation occupational healthcare units need to update their knowledge and understanding of these changes. In this regard, AMEs have a requirement for continuous training; these training sessions should cover the identification and monitoring of PTSD and assessments in terms of airworthiness.

## Limitations and practical applications

The major limitation in this review is that there is a lack of published literature on aircraft turbulence-related incidents and the association with PTSD. Additionally, there is very little research on PTSD related to aircraft incidents and crashes. This dearth of data makes it challenging to estimate the prevalence of PTSD associated with aviation accidents. Regardless, as a practical application we have highlighted the need to follow-up aviation personnel after severe turbulence. This review does not cover the mental trauma that has occurred to passengers onboard and their loved ones, ground personnel, air traffic controllers, rescue personnel and forensic personnel.
