# Supplementary material for: Assessment policy of post-traumatic stress disorder in aviation and its practical application using turbulence-triggered trauma as an example
Source: Front Public Health. 2025 Mar 18;13:1505004. doi: 10.3389/fpubh.2025.1505004 (PMC11959024; doi:10.3389/fpubh.2025.1505004)
Supplement: Supplementary file 1 [file Data_Sheet_1.PDF]

Appendix 1. DSM-5 PTSD Diagnostic Criteria (17)

**DSM-5 based PTSD Diagnostic Criteria**
Exposure to serious injury, actual or threatened death
Intrusions, Avoidance, Changes in cognition and mood, Arousal and reactivity
Duration over 1 month
Significant distress of functional impairment
Event caused physiological effects (f. ex.not due to medication)
